# 2-Hydr­oxy-*N*′-(4-isopropyl­cyclo­hexyl­carbon­yl)-3-methyl­benzohydrazide

**DOI:** 10.1107/S160053680900556X

**Published:** 2009-02-21

**Authors:** Tian-Pin Shu, Jun-Long Wen, Su-Zhen Chen, Ke-Wei Lei

**Affiliations:** a State Key Laboratory Base of Novel Functional Materials and Preparation Science, Institute of Solid Materials Chemistry, Faculty of Materials Science and Chemical Engineering, Ningbo University, Ningbo 315211, People’s Republic of China

## Abstract

The crystal structure of the title compound, C_18_H_26_N_2_O_3_, is stabilized by inter­molecular N—H⋯O and O—H⋯O hydrogen bonds. One of the methyl groups is disordered with occupancies of 0.51 (3):0.49 (3).

## Related literature

For the properties of metallocrowns, see: Alexiou *et al.* (2002 ▶); Gaynor *et al*. (2002 ▶); Lah & Pecoraro (1989 ▶); Lehaire *et al.* (2002 ▶); Liu *et al.* (2001 ▶, 2008 ▶); Saalfrank *et al.* (2001 ▶).
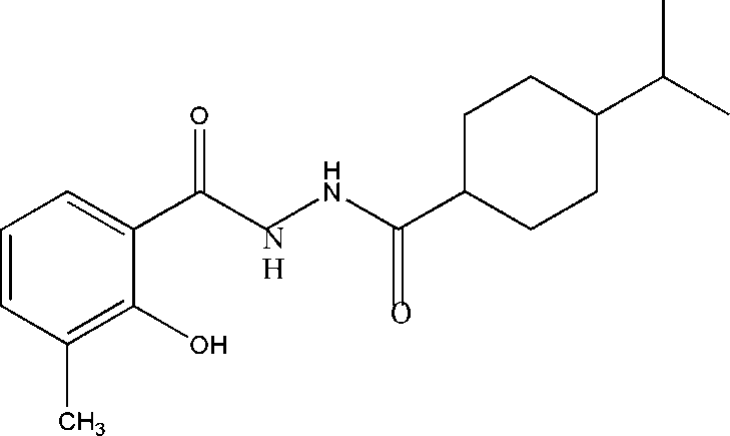


## Experimental

### 

#### Crystal data

C_18_H_26_N_2_O_3_
*M* *_r_* = 318.41
Monoclinic, 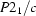

*a* = 16.193 (5) Å
*b* = 16.194 (5) Å
*c* = 6.856 (2) Å
β = 97.892 (4)°
*V* = 1780.8 (9) Å^3^
*Z* = 4
Mo *K*α radiation
μ = 0.08 mm^−1^
*T* = 296 K
0.43 × 0.26 × 0.22 mm

#### Data collection

Bruker APEXII CCD area-detector diffractometer
Absorption correction: none *T*_min_ = 0.970, *T*_max_ = 0.983
8728 measured reflections
6453 independent reflections
3925 reflections with *I* > 2σ(*I*)
*R*_int_ = 0.025

#### Refinement

*R*[*F* ^2^ > 2σ(*F* ^2^)] = 0.080
*wR*(*F* ^2^) = 0.211
*S* = 1.05
6453 reflections
217 parameters
H-atom parameters constrained
Δρ_max_ = 0.40 e Å^−3^
Δρ_min_ = −0.60 e Å^−3^

### 

Data collection: *APEX2* (Bruker, 2007 ▶); cell refinement: *SAINT* (Bruker, 2007 ▶); data reduction: *SAINT*; program(s) used to solve structure: *SHELXS97* (Sheldrick, 2008 ▶); program(s) used to refine structure: *SHELXL97* (Sheldrick, 2008 ▶); molecular graphics: *SHELXTL* (Sheldrick, 2008 ▶); software used to prepare material for publication: *SHELXTL*.

## Supplementary Material

Crystal structure: contains datablocks global, I. DOI: 10.1107/S160053680900556X/jh2073sup1.cif

Structure factors: contains datablocks I. DOI: 10.1107/S160053680900556X/jh2073Isup2.hkl

Additional supplementary materials:  crystallographic information; 3D view; checkCIF report

## Figures and Tables

**Table 1:** Hydrogen-bond geometry (Å, °)

*D*—H⋯*A*	*D*—H	H⋯*A*	*D*⋯*A*	*D*—H⋯*A*
N1—H1*A*⋯O2^i^	0.86	2.05	2.821 (4)	149
O1—H1*B*⋯O3	0.82	1.92	2.636 (4)	145
N2—H2*A*⋯O3^ii^	0.86	2.10	2.898 (4)	154

Symmetry codes: (i) 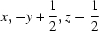; (ii) 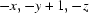.

**Fig. 1.**
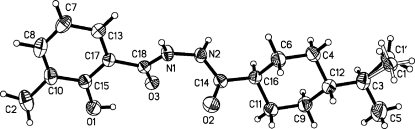
The structure of (I), showing 30% probability displacement ellipsoids and the atom-numbering scheme. [symmetry code: (i) –X, 0.5+Y, 0.5-Z].

**Fig. 2.**
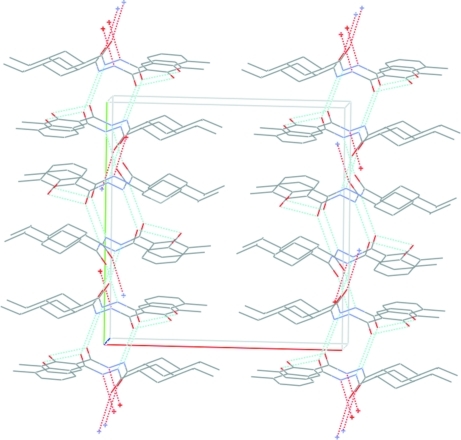
Packing diagram of (I). hydrogen bonds are shown as dashed lines.

## supplementary crystallographic information

### Comment

Metallacrowns are important compounds. Because of their potentially unique properties (Alexiou *et al.*, 2002; Gaynor *et al.*, 2002; Lah & Pecoraro, 1989; Lehaire *et al.*, 2002; Liu *et al.*, 2001; Saalfrank *et al.*, 2001), they have gained increasing attention over the past decade. These compounds can be readily assembled using a trianionic pentadentate ligand,*N*-acylsalicylhydrazide,having a trivalent octahedral metal ion. The size of the metallacrown can be controlled by modifying the close-contact interaction between the *N*-acyl residues of the ligands (Liu *et al.*, 2008). We now report structure of a designed pentadentate ligand, *N*-4-isoPropylcyclohexyl-3-methyl-salicylhydrazide.

A view of the title structure is illustrated in Fig.1. Because of C1 splited into C1 and C1', It made the *U*_eq_ of neighbor atoms lower or larger than usual *U*_eq_. The molecular conformation is characterized by N—H···O hydrogen bonds and the crystal packing is stabilized by N—H···O and O—H···O hydrogen bonds(Fig.2).

### Experimental

Trimethylaceto chloride (6.025 g, 50.0 mmol) was added to 50 ml chloroform solution of 4-isoPropylcyclohexyl acid with an external ice-water bath and triethylamine(5.200 g, 50.0 mmol). stirred for about 30 min slowly warmed to ambient temperature. To the above solution, 3-methyl-salicylhydrazide (7.636 g, 46.0 mmol)was added and stirred for 30 min. A white suspension began to appear after a while.the resulting white precipitate was filtered and rinsed with chloroform and diethyl ether. The title compound was recrystallized from methanol solution.

### Refinement

All H atoms were placed in geometrically idealized positions and constrained to ride on their parent atoms (C—H = 0.93%A; N—H = 0.86 Å; O—H = 0.82 Å) and *U*_iso_(H) values weren taken to be equal to 1.2 *U*_eq_(C, N) and 1.5Ueq(O). The C1 atom is disordered. Due to C12 is bonded to C3, which is not disordered. It has a smaller Ueq than other atoms, and thus has less freedom of movement. The larger than normal range of thermal motion is mostly due to the difference between the disordered group and the other atoms which are not disordered. The splited atom was dealed in the .ins file. the C1 atom is splited into C1 and C1', each of which has a half share. Then refinement, anisotropic refinement to convergence use the least-squares method.

### Crystal data

**Table:**

C_18_H_26_N_2_O_3_	*F*(000) = 688
*M**_r_* = 318.41	*D*_x_ = 1.188 Mg m^−^^3^
Monoclinic, *P*2_1_/*c*	Mo *K*α radiation, λ = 0.71073 Å
Hall symbol: -P 2ybc	Cell parameters from 5099 reflections
*a* = 16.193 (5) Å	θ = 2.5–26.2°
*b* = 16.194 (5) Å	µ = 0.08 mm^−^^1^
*c* = 6.856 (2) Å	*T* = 296 K
β = 97.892 (4)°	Block, colourless
*V* = 1780.8 (9) Å^3^	0.43 × 0.26 × 0.22 mm
*Z* = 4	

### Data collection

**Table:**

Bruker APEXII CCD area-detector diffractometer	3925 reflections with *I* > 2σ(*I*)
Radiation source: fine-focus sealed tube	*R*_int_ = 0.025
graphite	θ_max_ = 32.5°, θ_min_ = 1.8°
φ and ω scans	*h* = −16→24
8728 measured reflections	*k* = −17→24
6453 independent reflections	*l* = −7→10

### Refinement

**Table:**

Refinement on *F*^2^	Secondary atom site location: difference Fourier map
Least-squares matrix: full	Hydrogen site location: inferred from neighbouring sites
*R*[*F*^2^ > 2σ(*F*^2^)] = 0.080	H-atom parameters constrained
*wR*(*F*^2^) = 0.211	*w* = 1/[σ^2^(*F*_o_^2^) + (0.0912*P*)^2^ + 2.6628*P*] where *P* = (*F*_o_^2^ + 2*F*_c_^2^)/3
*S* = 1.05	(Δ/σ)_max_ = 0.001
6453 reflections	Δρ_max_ = 0.40 e Å^−^^3^
217 parameters	Δρ_min_ = −0.60 e Å^−^^3^
0 restraints	Extinction correction: *SHELXL97* (Sheldrick, 2008), Fc^*^=kFc[1+0.001xFc^2^λ^3^/sin(2θ)]^-1/4^
Primary atom site location: structure-invariant direct methods	Extinction coefficient: 0.016 (5)

### Special details

**Table:**

Geometry. All e.s.d.'s (except the e.s.d. in the dihedral angle between two l.s. planes) are estimated using the full covariance matrix. The cell e.s.d.'s are taken into account individually in the estimation of e.s.d.'s in distances, angles and torsion angles; correlations between e.s.d.'s in cell parameters are only used when they are defined by crystal symmetry. An approximate (isotropic) treatment of cell e.s.d.'s is used for estimating e.s.d.'s involving l.s. planes.
Refinement. Refinement of *F*^2^ against ALL reflections. The weighted *R*-factor *wR* and goodness of fit *S* are based on *F*^2^, conventional *R*-factors *R* are based on *F*, with *F* set to zero for negative *F*^2^. The threshold expression of *F*^2^ > σ(*F*^2^) is used only for calculating *R*-factors(gt) *etc*. and is not relevant to the choice of reflections for refinement. *R*-factors based on *F*^2^ are statistically about twice as large as those based on *F*, and *R*- factors based on ALL data will be even larger.

### Fractional atomic coordinates and isotropic or equivalent isotropic displacement parameters (Å^2^)

**Table:**

	*x*	*y*	*z*	*U*_iso_*/*U*_eq_	Occ. (<1)
O1	0.26649 (17)	0.4109 (2)	0.1034 (4)	0.0739 (9)	
H1B	0.2257	0.4290	0.1481	0.111*	
O2	−0.02604 (18)	0.29519 (17)	0.2523 (4)	0.0721 (9)	
O3	0.10747 (16)	0.44725 (15)	0.0967 (4)	0.0527 (7)	
N1	0.01874 (18)	0.36122 (18)	−0.0854 (5)	0.0512 (9)	
H1A	0.0117	0.3227	−0.1724	0.061*	
N2	−0.04755 (18)	0.38761 (18)	0.0058 (5)	0.0513 (8)	
H2A	−0.0782	0.4274	−0.0462	0.062*	
C1	−0.458 (2)	0.3768 (18)	0.388 (6)	0.056 (4)	0.49 (3)
H1C	−0.4868	0.4194	0.3090	0.084*	0.49 (3)
H1D	−0.4921	0.3564	0.4804	0.084*	0.49 (3)
H1E	−0.4444	0.3325	0.3043	0.084*	0.49 (3)
C1'	−0.454 (2)	0.3490 (17)	0.412 (6)	0.056 (4)	0.51 (3)
H1'A	−0.5058	0.3693	0.4471	0.084*	0.51 (3)
H1'B	−0.4431	0.2950	0.4669	0.084*	0.51 (3)
H1'C	−0.4580	0.3460	0.2709	0.084*	0.51 (3)
C2	0.3959 (3)	0.3661 (4)	−0.0978 (10)	0.106 (2)	
H2B	0.4317	0.3483	−0.1904	0.159*	
H2C	0.4051	0.3319	0.0176	0.159*	
H2D	0.4082	0.4225	−0.0618	0.159*	
C3	−0.3787 (3)	0.4114 (4)	0.4969 (7)	0.0834 (15)	
H3A	−0.3790	0.4699	0.4600	0.100*	
C4	−0.2983 (3)	0.3734 (3)	0.2158 (6)	0.0757 (14)	
H4A	−0.3073	0.4287	0.1626	0.091*	
H4B	−0.3448	0.3394	0.1591	0.091*	
C5	−0.3791 (4)	0.4114 (5)	0.7111 (8)	0.119 (2)	
H5A	−0.4301	0.4355	0.7407	0.179*	
H5B	−0.3326	0.4430	0.7733	0.179*	
H5C	−0.3748	0.3556	0.7591	0.179*	
C6	−0.2185 (2)	0.3391 (3)	0.1563 (6)	0.0697 (12)	
H6A	−0.2224	0.3394	0.0138	0.084*	
H6B	−0.2116	0.2824	0.2005	0.084*	
C7	0.2006 (3)	0.3240 (3)	−0.4629 (6)	0.0659 (12)	
H7A	0.1872	0.3061	−0.5923	0.079*	
C8	0.2827 (3)	0.3312 (3)	−0.3797 (7)	0.0722 (13)	
H8A	0.3242	0.3168	−0.4546	0.087*	
C9	−0.2194 (3)	0.4244 (3)	0.5271 (6)	0.0668 (12)	
H9A	−0.2261	0.4820	0.4885	0.080*	
H9B	−0.2155	0.4219	0.6694	0.080*	
C10	0.3056 (3)	0.3589 (3)	−0.1909 (7)	0.0667 (12)	
C11	−0.1385 (2)	0.3919 (3)	0.4654 (6)	0.0639 (11)	
H11A	−0.0927	0.4272	0.5202	0.077*	
H11B	−0.1278	0.3368	0.5182	0.077*	
C12	−0.2964 (2)	0.3765 (3)	0.4368 (6)	0.0594 (11)	
H12A	−0.2903	0.3197	0.4858	0.071*	
C13	0.1385 (3)	0.3435 (2)	−0.3521 (5)	0.0519 (10)	
H13A	0.0829	0.3375	−0.4057	0.062*	
C14	−0.0656 (2)	0.3531 (2)	0.1735 (5)	0.0489 (9)	
C15	0.2426 (2)	0.3815 (2)	−0.0817 (6)	0.0525 (10)	
C16	−0.1424 (2)	0.3893 (2)	0.2434 (5)	0.0491 (9)	
H16A	−0.1494	0.4459	0.1934	0.059*	
C17	0.1590 (2)	0.3722 (2)	−0.1597 (5)	0.0438 (9)	
C18	0.0941 (2)	0.3959 (2)	−0.0379 (5)	0.0432 (9)	

### Atomic displacement parameters (Å^2^)

**Table:**

	*U*^11^	*U*^22^	*U*^33^	*U*^12^	*U*^13^	*U*^23^
O1	0.0567 (17)	0.097 (2)	0.0653 (19)	−0.0119 (16)	0.0002 (14)	−0.0117 (16)
O2	0.078 (2)	0.0557 (17)	0.084 (2)	0.0235 (15)	0.0142 (16)	0.0192 (15)
O3	0.0640 (16)	0.0435 (14)	0.0507 (15)	−0.0001 (12)	0.0089 (12)	−0.0073 (12)
N1	0.0474 (18)	0.0430 (17)	0.065 (2)	−0.0004 (14)	0.0150 (15)	−0.0112 (14)
N2	0.0483 (18)	0.0446 (17)	0.063 (2)	0.0103 (14)	0.0156 (15)	0.0052 (15)
C1	0.052 (4)	0.062 (15)	0.054 (8)	0.001 (11)	0.007 (5)	−0.002 (10)
C1'	0.052 (4)	0.062 (15)	0.054 (8)	0.001 (11)	0.007 (5)	−0.002 (10)
C2	0.049 (3)	0.133 (5)	0.137 (5)	−0.003 (3)	0.021 (3)	0.000 (4)
C3	0.066 (3)	0.111 (4)	0.078 (3)	0.003 (3)	0.026 (2)	−0.003 (3)
C4	0.052 (2)	0.114 (4)	0.060 (3)	0.003 (2)	0.005 (2)	−0.012 (2)
C5	0.089 (4)	0.182 (7)	0.091 (4)	−0.019 (4)	0.029 (3)	−0.020 (4)
C6	0.055 (2)	0.094 (3)	0.060 (2)	−0.002 (2)	0.0059 (19)	−0.022 (2)
C7	0.091 (3)	0.052 (2)	0.060 (2)	−0.007 (2)	0.028 (2)	−0.0087 (19)
C8	0.077 (3)	0.061 (3)	0.087 (3)	0.001 (2)	0.041 (3)	−0.007 (2)
C9	0.066 (3)	0.080 (3)	0.056 (2)	−0.004 (2)	0.013 (2)	−0.015 (2)
C10	0.052 (2)	0.064 (3)	0.087 (3)	−0.0014 (19)	0.021 (2)	0.004 (2)
C11	0.055 (2)	0.079 (3)	0.057 (2)	−0.007 (2)	0.0021 (19)	−0.006 (2)
C12	0.059 (2)	0.062 (2)	0.058 (2)	−0.0005 (19)	0.0116 (19)	0.0021 (19)
C13	0.062 (2)	0.042 (2)	0.052 (2)	−0.0038 (17)	0.0107 (18)	−0.0033 (16)
C14	0.050 (2)	0.039 (2)	0.058 (2)	0.0026 (17)	0.0056 (17)	0.0026 (17)
C15	0.051 (2)	0.049 (2)	0.058 (2)	−0.0037 (17)	0.0101 (18)	0.0030 (17)
C16	0.052 (2)	0.043 (2)	0.053 (2)	0.0033 (16)	0.0103 (17)	0.0060 (16)
C17	0.049 (2)	0.0339 (18)	0.050 (2)	−0.0022 (15)	0.0113 (16)	0.0022 (15)
C18	0.052 (2)	0.0313 (17)	0.046 (2)	0.0023 (15)	0.0077 (16)	0.0037 (16)

### Geometric parameters (Å, °)

**Table:**

O1—C15	1.360 (5)	C4—H4B	0.9700
O1—H1B	0.8200	C5—H5A	0.9600
O2—C14	1.220 (4)	C5—H5B	0.9600
O3—C18	1.239 (4)	C5—H5C	0.9600
N1—C18	1.342 (5)	C6—C16	1.527 (6)
N1—N2	1.382 (4)	C6—H6A	0.9700
N1—H1A	0.8600	C6—H6B	0.9700
N2—C14	1.346 (5)	C7—C13	1.376 (6)
N2—H2A	0.8600	C7—C8	1.378 (7)
C1—C3	1.50 (4)	C7—H7A	0.9300
C1—H1C	0.9600	C8—C10	1.372 (6)
C1—H1D	0.9600	C8—H8A	0.9300
C1—H1E	0.9600	C9—C11	1.525 (6)
C1'—C3	1.63 (4)	C9—C12	1.526 (6)
C1'—H1'A	0.9600	C9—H9A	0.9700
C1'—H1'B	0.9600	C9—H9B	0.9700
C1'—H1'C	0.9600	C10—C15	1.394 (6)
C2—C10	1.518 (7)	C11—C16	1.515 (5)
C2—H2B	0.9600	C11—H11A	0.9700
C2—H2C	0.9600	C11—H11B	0.9700
C2—H2D	0.9600	C12—H12A	0.9800
C3—C5	1.469 (7)	C13—C17	1.395 (5)
C3—C12	1.554 (6)	C13—H13A	0.9300
C3—H3A	0.9800	C14—C16	1.511 (5)
C4—C12	1.512 (6)	C15—C17	1.395 (5)
C4—C6	1.514 (6)	C16—H16A	0.9800
C4—H4A	0.9700	C17—C18	1.479 (5)
			
C15—O1—H1B	109.5	H6A—C6—H6B	107.9
C18—N1—N2	119.8 (3)	C13—C7—C8	119.2 (4)
C18—N1—H1A	120.1	C13—C7—H7A	120.4
N2—N1—H1A	120.1	C8—C7—H7A	120.4
C14—N2—N1	122.1 (3)	C10—C8—C7	122.6 (4)
C14—N2—H2A	118.9	C10—C8—H8A	118.7
N1—N2—H2A	118.9	C7—C8—H8A	118.7
C3—C1—H1C	109.5	C11—C9—C12	113.4 (3)
C3—C1—H1D	109.5	C11—C9—H9A	108.9
C3—C1—H1E	109.5	C12—C9—H9A	108.9
C3—C1'—H1'A	109.5	C11—C9—H9B	108.9
C3—C1'—H1'B	109.5	C12—C9—H9B	108.9
H1'A—C1'—H1'B	109.5	H9A—C9—H9B	107.7
C3—C1'—H1'C	109.5	C8—C10—C15	118.0 (4)
H1'A—C1'—H1'C	109.5	C8—C10—C2	122.8 (4)
H1'B—C1'—H1'C	109.5	C15—C10—C2	119.2 (4)
C10—C2—H2B	109.5	C16—C11—C9	111.7 (3)
C10—C2—H2C	109.5	C16—C11—H11A	109.3
H2B—C2—H2C	109.5	C9—C11—H11A	109.3
C10—C2—H2D	109.5	C16—C11—H11B	109.3
H2B—C2—H2D	109.5	C9—C11—H11B	109.3
H2C—C2—H2D	109.5	H11A—C11—H11B	107.9
C5—C3—C1	112.2 (16)	C4—C12—C9	109.0 (4)
C5—C3—C12	112.8 (4)	C4—C12—C3	112.2 (4)
C1—C3—C12	115.8 (16)	C9—C12—C3	112.9 (4)
C5—C3—C1'	104.6 (14)	C4—C12—H12A	107.5
C1—C3—C1'	17.0 (13)	C9—C12—H12A	107.5
C12—C3—C1'	107.9 (14)	C3—C12—H12A	107.5
C5—C3—H3A	104.9	C7—C13—C17	120.1 (4)
C1—C3—H3A	104.9	C7—C13—H13A	119.9
C12—C3—H3A	104.9	C17—C13—H13A	119.9
C1'—C3—H3A	121.9	O2—C14—N2	122.3 (3)
C12—C4—C6	112.4 (4)	O2—C14—C16	124.3 (3)
C12—C4—H4A	109.1	N2—C14—C16	113.3 (3)
C6—C4—H4A	109.1	O1—C15—C10	117.2 (4)
C12—C4—H4B	109.1	O1—C15—C17	122.1 (3)
C6—C4—H4B	109.1	C10—C15—C17	120.6 (4)
H4A—C4—H4B	107.8	C14—C16—C11	114.0 (3)
C3—C5—H5A	109.5	C14—C16—C6	108.9 (3)
C3—C5—H5B	109.5	C11—C16—C6	109.1 (3)
H5A—C5—H5B	109.5	C14—C16—H16A	108.2
C3—C5—H5C	109.5	C11—C16—H16A	108.2
H5A—C5—H5C	109.5	C6—C16—H16A	108.2
H5B—C5—H5C	109.5	C15—C17—C13	119.4 (3)
C4—C6—C16	111.9 (4)	C15—C17—C18	118.9 (3)
C4—C6—H6A	109.2	C13—C17—C18	121.7 (3)
C16—C6—H6A	109.2	O3—C18—N1	121.5 (3)
C4—C6—H6B	109.2	O3—C18—C17	122.0 (3)
C16—C6—H6B	109.2	N1—C18—C17	116.5 (3)

### Hydrogen-bond geometry (Å, °)

**Table:**

*D*—H···*A*	*D*—H	H···*A*	*D*···*A*	*D*—H···*A*
N1—H1A···O2^i^	0.86	2.05	2.821 (4)	149
O1—H1B···O3	0.82	1.92	2.636 (4)	145
N2—H2A···O3^ii^	0.86	2.10	2.898 (4)	154

Symmetry codes: (i) *x*, −*y*+1/2, *z*−1/2;  (ii) −*x*, −*y*+1, −*z*.
