# 2-Hydr­oxy-*N*′-(4-isopropyl­cyclo­hexyl­carbon­yl)-3-methyl­benzohydrazide. Corrigendum

**DOI:** 10.1107/S1600536809017863

**Published:** 2009-05-23

**Authors:** Tian-Pin Shu, Yu Zhou, Jun-Long Wen, Su-Zhen Chen, Ke-Wei Lei

**Affiliations:** a State Key Laboratory Base of Novel Functional Materials and Preparation Science, Institute of Solid Materials Chemistry, Faculty of Materials Science and Chemical Engineering, Ningbo University, Ningbo 315211, People’s Republic of China

## Abstract

The author list in the paper by Shu, Wen, Chen & Lei [*Acta Cryst.* (2009), E**65**, o575] is corrected.

## Abstract 2

Corrigendum to *Acta Cryst.* (2009), E**65**, o575.

In the paper by Shu, Wen, Chen & Lei [*Acta Cryst.* (2009), E**65**, o575], the author list was incorrect. The correct author list is given above.

